# Pathogenicity of a TW-Like Strain of Infectious Bronchitis Virus and Evaluation of the Protection Induced against It by a QX-Like Strain

**DOI:** 10.3389/fmicb.2016.01653

**Published:** 2016-10-18

**Authors:** Shi-hong Yan, Yang Chen, Jing Zhao, Gang Xu, Ye Zhao, Guo-zhong Zhang

**Affiliations:** Key Laboratory of Animal Epidemiology and Zoonoses, Ministry of Agriculture, College of Veterinary Medicine, China Agricultural UniversityBeijing, China

**Keywords:** avian infectious bronchitis virus, isolate, pathogenicity, vaccine, efficacy

## Abstract

Avian infectious bronchitis, a highly contagious disease caused by avian infectious bronchitis virus (IBV), is of considerable economic importance to the poultry industry. New IBV TW-like strains have increasingly emerged in China in recent years; hence, evaluating their pathogenicity and developing a specific vaccine to guard against their potential threat to the poultry industry is important. Here, we examined the pathogenicity of a TW-like IBV strain (GD), and evaluated the protective efficacy of the QX-like strain (JS) against GD in challenge infections in chickens. The results revealed that strain-GD-infected birds experienced severe respiratory signs, renal lesions, and 30–40% mortality. The GD virus had extensive tissue tropism, especially in the trachea, lungs, kidneys, and bursa of Fabricius, and was continuously shed via the respiratory tract and cloaca. The QX-like IBV strain JS is able to completely protect chickens from challenge with the TW-like IBV GD field strain, with no clinical signs or gross lesions, decreased tissue replication rates, lower ciliostasis score, and reduced virus shedding. These findings indicate that IBV GD is highly virulent, and that QX-like JS may serve as an effective vaccine against the threat posed by IBV TW-like viruses.

## Introduction

Infectious bronchitis (IB) is an acute, highly contagious respiratory, and urogenital disease caused by infections bronchitis virus (IBV). The IBV genome consists of a positive-stranded RNA of 27.6 kb in length, and the virus belongs to the *Gammacoronavirus* genus (*Coronaviridae* family, *Nidovirales* order; Cavanagh et al., 1992). The disease, which affects chickens of all ages, poses a major economic threat to the worldwide poultry industry because of poor weight gain and lost feeding efficiency in broilers, and reduced egg numbers and quality in egg-laying birds (Yu et al., 2001; Xu et al., 2007; Jackwood, 2012).

Currently, the main method of protecting chickens from IB is vaccination with both live and inactivated vaccines (Zhao et al., 2015). However, effective vaccination is undermined by rapidly acquired genetic changes in IBV (e.g., gene insertion, mutation, deletion, and reconstruction; Dolz et al., 2008; Kuo et al., 2010). Additionally, immunity against IBV affords a low degree of cross protection between different IBV serotypes (Cowen and Hitchner, 1975), thus confirming the results of molecular epidemiology studies on IBV (Han et al., 2011; Li et al., 2012). Because of the inaccuracy of the coronavirus RNA-dependent RNA polymerase and high frequency of homologous RNA recombination, the emergence of new variant strains, genotypes, and serotypes of IBV is continuously reported (Cavanagh et al., 1986). Existing vaccines for IB no longer provide protection and they tend to be ineffective against the epidemic IBV strains when new variant IBV isolates emerge or when new serotypes appear (Sun et al., 2011). Therefore, screening new live-attenuated vaccine strains based on new isolates is necessary to protect chickens against the common epidemic IBV strains.

A predominant IBV type, QX-like IBV, has been circulating in China since 1998 when the first variant of this type was reported (Wang et al., 1998; Zhao et al., 2014). Over time, we have seen a sustained increase in the QX-like genotype in China (from 11.7 to nearly 70% at present; Zhao et al., 2016). Concurrently, there have been increasing reports of QX-like cases in many other countries (Thailand, Zimbabwe, Korea, Denmark, France, Germany, Russia, Spain, and UK), where the viruses involved share sequence similarities with QX-like viruses (Domanskablicharz et al., 2006; Worthington and Jones, 2006; Worthington et al., 2008; Valastro et al., 2010; Krapež et al., 2011; Abro et al., 2012; Amin et al., 2012; Mo et al., 2013). However, since the first TW-like domestic strain CK/CH/LSD/05I was isolated in the Shandong Province of China, an increasing number of TW-like strains have also been isolated in mainland China (Liu et al., 2008), as confirmed by our previous findings (Xu et al., 2016).

Here, we determined the pathogenicity of the TW-like IBV GD strain. We also evaluated the protective efficacy induced by QX-like strain JS against the TW-like field strain GD by observing the clinical signs and gross lesions, and by analysis of the virus distribution, virus shedding, and tracheal ciliary activity in experimental chickens. Our results suggest that TW-like IBV GD is highly virulent, and that QX-like JS may provide effective vaccine protection against TW-like IBV viruses.

## Materials and Methods

### Viruses

The IBV TW-like GD strain was isolated from IB-affected chickens in the Guangdong Province of China. These chickens had been vaccinated with IBV Mass-type vaccines, and were affected by respiratory and renal disease. The complete genome of the IBV strain GD was sequenced in our previous study (Xu et al., 2016). The IBV QX-like JS strain, which has naturally low virulence, was isolated from IB-affected chickens in the Jiangsu Province of China in 2012. JS and GD viruses were each purified by limiting dilution, and purified allantoic fluid-containing JS or GD strains was titrated by inoculation into the allantoic sacs of 10-day-old specific-pathogen-free (SPF) embryonated eggs. The median embryo infectious doses (EID_50_) of the JS and GD strains were calculated as 10^8.02^ EID_50_/mL and 10^8.03^ EID_50_/mL, respectively.

### Animals and Ethics Statement

Specific-pathogen-free white leghorn chickens were purchased from the Beijing Merial Vital Laboratory Animal Technology Co., Ltd, China. All the animals used in this study were cared for in accordance with established guidelines, and the experimental protocols (including the possibility of animal death without euthanasia) were approved by the Animal Welfare and Ethical Censor Committee of China Agricultural University (Approval No. SYXK 2013-0013).

### Pathogenicity Testing

#### Grouping and Sampling

Fifty 3-week-old SPF chickens were assigned randomly to four groups. Groups A and B were used to observe chicken mortality (*n* = 10 each). Groups C and D were used for sampling (*n* = 15 each). All the chickens were maintained in isolators under positive pressure in air-conditioned rooms, and food and water were provided *ad libitum*. Groups A and C were inoculated with 200 μl of allantoic fluid containing 10^6.00^ EID_50_ of the GD virus via the intranasal and intraocular routes. Groups B and D were maintained as uninfected controls. All the chickens were monitored daily for 14 days. Three chicks from groups C and D were sacrificed at 3, 5, 7, and 10 days post-challenge (dpc). Gross lesions and tracheal ciliary activity were evaluated in the birds. Tissue samples from the trachea, lungs, spleen, glandular stomach, kidneys, and bursa of Fabricius were collected. Some birds died during the clinical observation period, and their tissue samples were also collected. The tissue samples were placed in 10% neutral formalin for histology, or stored at -80°C for viral RNA detection using reverse transcription–polymerase chain reaction (RT-PCR).

#### Viral Shedding Detection

Oral and cloaca swabs were randomly collected at 5 and 7 dpc from 10 birds in challenge group C and five birds in control group D to detect viral shedding via RT-PCR. Viral RNA was extracted with TRIzol^®^ Reagent (Invitrogen, Carlsbad, CA, USA) according to the manufacturer’s instructions. RT was performed at 37°C for 1 h with 3 μg of total RNA, 1 μl of random hexamer primers (500 μg/ml; Promega, Madison, WI, USA), and 0.5 μl of M-MLV reverse transcriptase (200 U/μl; Promega). PCR was performed in a 20 μl reaction volume containing 10 μl of Taq SuperMix (TransGen, Beijing, China), 10 pmol of each primer, 100 ng of cDNA as the template, and 7 μl of water. Thermal cycling was performed at 95°C for 5 min, followed by 30 cycles of denaturation (95°C, 45 s), annealing (55°C, 45 s), and polymerization (72°C, 1 min), and a final extension step was performed at 72°C for 10 min. A pair of primers (forward: 5′- GTTGTTGTGGTGTTGTTGTG-3′; reverse: 5′-CAAGGGTGCAATTTGTCT-3′) that amplified an 800-bp fragment of the IBV N gene was used in the PCR. The amplified fragments were detected by 1.0% agarose gel electrophoresis.

#### Viral RNA Detection in Infected Tissue Samples

Total RNA was extracted from the trachea, lungs, spleen, glandular stomach, kidneys, and bursa of Fabricius with TRIzol^®^ Reagent (Invitrogen) following the manufacturer’s protocol. To transcribe the first cDNA strand, a mixture containing 1 μl of random primers (500 μg/ml), 2.0 μl of dNTPs (2.5 mM), 1.5 μl of RNasin^®^ (50 U/μl), 0.5 μl of M-MLV reverse transcriptase (10 U/μl), and 4 μl of the RNA template was incubated at 37°C for 60 min, and then at 72°C for 5 min. The product was used for PCR, as described above.

#### Inhibition of Ciliary Activity

To evaluate tracheal ciliostasis, three sections of the upper, middle, and lower parts of the trachea (nine rings per bird) were analyzed. The rings were placed in 96-well plates with Eagle’s culture medium containing 10% fetal bovine serum. They were then examined by inverted light microscopy at a magnification of 400× to determine the degree of integrity and the preservation of ciliary movement in the tracheal epithelial cells. A score of 0 was given if the cilia in the complete tracheal section showed movement; a score of 1 was given if 75–100% of the cilia in the tracheal section showed movement; a score of 2 was given if 50–75% of the cilia in the tracheal section showed movement; a score of 3 was given if 25–50% of the cilia in the tracheal section showed movement; and a score of 4 was given if <25% of the cilia in the tracheal section showed movement or there was no movement at all. The average ciliostasis score was calculated for each group.

#### Histopathology

Tissue samples from the trachea, lungs, spleen, glandular stomach, kidneys, and bursa of Fabricius were fixed in 10% neutral formalin for 48 h at room temperature. These samples were processed routinely, embedded in paraffin wax, and cut into 5 μm sections. The sections were stained with hematoxylin and eosin (H&E) and examined for lesions with light microscopy.

### Efficacy of the JS Strain against Challenge Infections with the GD Strain

Forty-eight 6-week-old SPF chickens were randomly divided into three groups of 16 chickens each and then housed in different isolators. Group JS-GD was vaccinated with 200 μL of 10^7.00^ EID_50_ of the JS strain by intranasal and intraocular administration. The group control and control-GD group were inoculated with the same volume of sterile saline by the same method. Twenty-one days post-inoculation (dpi), groups JS-GD, and control-GD were challenged with 200 μL of the GD strain containing 10^6.00^ EID_50_ by intranasal and ocular routes. The group control was inoculated with sterile saline using the same method. Birds from all groups were housed with consistent conditions.

All chicks were observed daily for clinical signs attributable to IB infection for 14 days after challenge. Three chicks from each group were killed humanely and dissected at 5 and 7 dpc. When chicks died during the observation period autopsies were performed in a timely manner. External and internal exceptional situations were recorded in detail and samples of trachea, lungs, kidneys, spleen, glandular stomach, and bursa of Fabricius were collected for virus detection by RT-PCR. Additionally, ten laryngeal swabs from each group were collected for virus detection by RT-PCR by random sampling at 5 and 7 dpc. Tracheal ciliary activity was evaluated at 5 and 7 dpc. RT-PCR and tracheal ciliostasis evaluation were performed as described in the pathogenicity tests. Blood samples from ten chicks in each group were collected at 21 dpi and 14 dpc for IBV antibody analysis using an enzyme-linked immunosorbent assay (ELISA; IDEXX Laboratories, Westbrook, ME, USA).

### Statistical Analyses

Data were analyzed using an unpaired *t*-test in GraphPad Prism version 6.0 for Windows to obtain a statistical analysis of the differences between group control-GD and JS-GD in the protective efficacy test. For the ciliary activity inhibition test, the data collected were analyzed using two-way ANOVA to see whether there was any significant difference between the different groups. Multiple comparisons among the groups were performed using Tukey’s multiple comparison test. Statistical significance was considered as follows: significant at ^∗^*P* ≤ 0.05; highly significant at ^∗∗^*P* ≤ 0.01; and very highly significant at ^∗∗∗^*P* ≤ 0.001.

## Results

### Pathogenicity Testing

#### Clinical Observations

**FIGURE 1:**
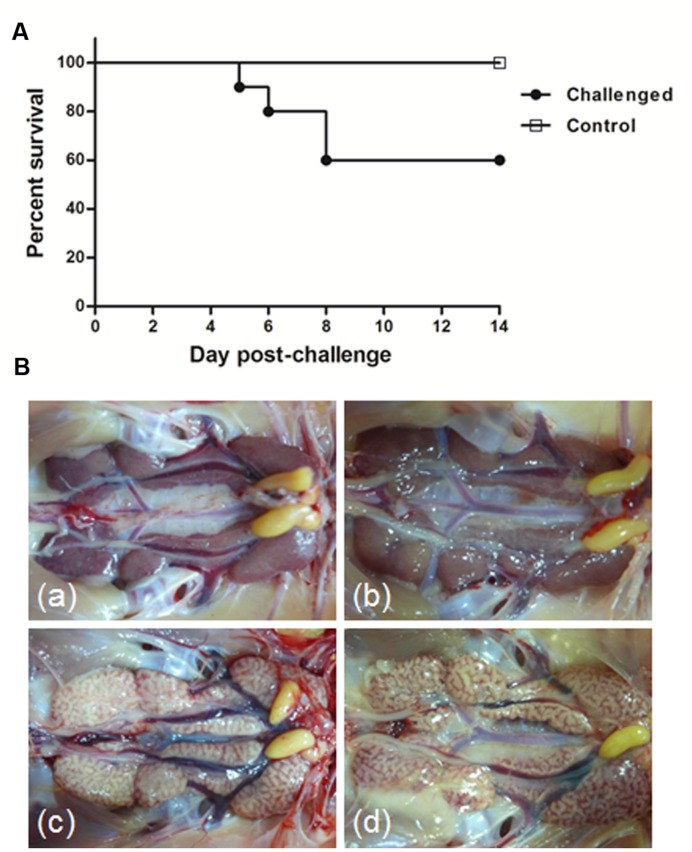
**Percentage survival (A) and gross lesions in the kidneys (B) of 3-week-old specific-pathogen-free (SPF) chickens challenged with infectious bronchitis virus (IBV) strain GD.** (a) Kidney tissue from an uninfected control chicken. (b,c) Obvious kidney enlargement and urate deposition at 5 and 7 days post-challenge (dpc), respectively, in a chick infected with IBV strain GD. (d) Obvious pale enlarged kidney with urate deposits from a chick infected with strain GD that died at 6 dpc.

Three-week-old SPF chickens challenged with the GD strain showed clinical signs of infection as early as 3 dpc, and these signs persisted until 10 dpc. The diseased chicks showed signs of coughing, sneezing, tracheal and bronchiolar rales, listlessness, huddling, and ruffled feathers. Four birds in the GD-inoculated group A died during the experiment, beginning at 5 dpc and peaking at 8 dpc, and the mortality rate reached 40% (**Figure 1A**). The birds in the control groups were alert and active during the experiment.

#### Gross Lesions

At necropsy, obvious lesions were detected in the respiratory tract, lungs, kidneys, and bursa of Fabricius in the chickens infected with the GD strain, including punctate hemorrhage in the throat and trachea and catarrhal exudate in the nasal cavity. The main manifestations in the sampled chicken were swollen kidneys, with urate deposits frequently observed in the tubules and ureters (‘spotted kidney’) at 5 and 7 dpc, and in the dead chickens at 6 dpc (**Figure 1B**, b–d). The bursa of Fabricius showed a heavy exudate of mucus and was in some cases atrophied. No obvious lesions were identified in any other organs in the infection-challenged group or in any birds that recovered from infection with strain GD. No gross lesions were observed in any birds in the control group (**Figure 1B**, a).

#### Histopathology

**FIGURE 2:**
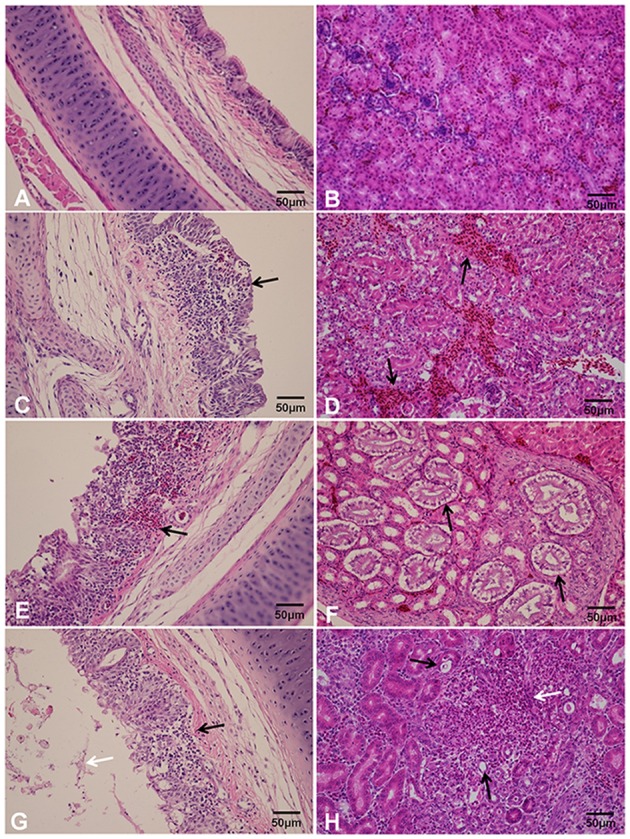
**Histopathological changes in the trachea (A,C,E,G) and kidneys (B,D,F,H) of 3-week-old SPF chickens challenged with IBV strain GD. (A)** No obvious tracheal lesions were seen in the control group; **(B)** no obvious kidney lesions were found in the control group; **(C)** black arrow indicates the thickening of the lamina propria of the tracheal mucosa and inflammatory cell infiltration; **(D,E)** hemorrhage; **(F)** separation of the kidney tubules from the basilar membrane; **(G)** white arrow indicates extensive drop out, degeneration, and necrosis of ciliated epithelial cells; **(H)** black arrows indicates necrosis of the kidney epithelial cells and white arrow indicates inflammatory cell infiltration.

Moderate to severe lesions were found predominantly between 5 and 7 dpc in the organs collected from the infection-challenged chickens. The chickens exposed to strain GD had the following lesions: thickening and hemorrhage of the lamina propria of the tracheal mucosa, inflammatory cell infiltrates, and varying degrees of epithelial desquamation and necrosis of the ciliated cells in the trachea at 3, 5, and 7 dpc, respectively (**Figures 2C,E,G**). Foci of necrosis, intense multifocal nephritis with lymphoplasmacytic infiltration, and mesenchyme dilation were scattered throughout the kidneys of the GD-inoculated chickens at 3, 5, and 7 dpc, respectively (**Figures 2D,F,H**). Hemorrhage, congestion, erythrocytes, and lymphocytes were frequently detected in the bronchial and air capillary lumina in the lungs. Epithelial cell necrosis and lymphocyte emptying were detected in the bursa. No IBV-related lesions were observed in the control birds (**Figures 2A,B**).

#### Inhibition of Ciliary Activity

Inhibition of ciliary activity in trachea was measured at 3, 5, 7, and 10 dpc. The infection-challenged group showed a maximum average ciliostasis score of approximately 4, whereas that of the control was <1. The difference in ciliostasis between the strain-GD-infected group and the control group was highly significant (*p* < 0.0001).

#### Viral Shedding

The viral shedding rate, as determined by RT-PCR, was 100% in the challenged group at 5 and 7 dpc. No viral RNA was detected in the control group.

#### Viral RNA Detection from Infected Tissue Samples

No viral RNA was detected in any tissues from the control group. In the infection-challenged group, the virus RNA was detected in the trachea, kidneys, and bursa of Fabricius at 3–10 dpc and in the lungs, spleen, and glandular stomach at 3–7 dpc. The proportion of virus-positive samples in the GD-infected group was 62.5% (45/72). **Table 1** shows the results for viral RNA detection for the infected groups and control groups.

**Table 1:** Viral RNA detection of sampled tissues in 3-week-old specific-pathogen-free (SPF) chickens challenged with infectious bronchitis virus (IBV) strain GD by reverse transcription–polymerase chain reaction (RT-PCR).

Organs	Control				Challenged			
	3 dpc^a^	5 dpc	7 dpc	10 dpc	3 dpc	5 dpc	7 dpc	10 dpc
Trachea	0/3^b^	0/3	0/3	0/3	3/3	3/3	1/3	2/3
Lung	0/3	0/3	0/3	0/3	1/3	2/3	3/3	0/3
Spleen	0/3	0/3	0/3	0/3	1/3	2/3	1/3	0/3
Forestomach	0/3	0/3	0/3	0/3	3/3	2/3	2/3	0/3
Kidney	0/3	0/3	0/3	0/3	2/3	3/3	3/3	2/3
Bursa of Fabricius	0/3	0/3	0/3	0/3	3/3	2/3	3/3	1/3

*^a^Day post-challenge.*

*^b^Number of positive birds/total number of birds sampled at each time point.*

### Efficacy of JS Strain against Challenge with GD Strain

#### Clinical Manifestations

**FIGURE 3:**
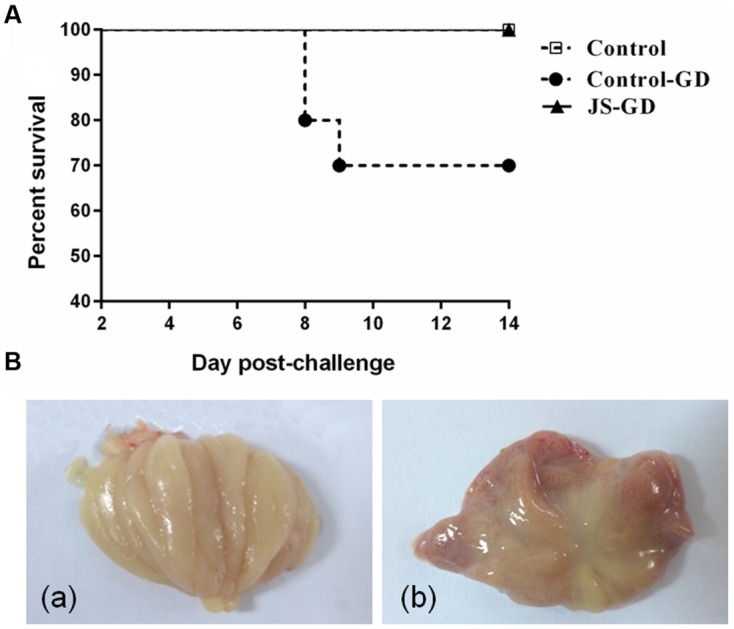
**Survival percentage (A) and gross lesions of bursa of Fabricius (B) in 9-week-old SPF chickens experimentally challenged with IBV GD strain.** (a) No obvious lesions were found in the bursa of Fabricius in group JS-GD. (b) Heavy exudates of flaxen mucus on the bursa of Fabricius in group GD.

In the control-GD group, four chicks showed signs of depression and tracheal and bronchiolar rales at 7 dpc. The mortality of the group control-GD was 30% during the 14-day observation period (**Figure 3A**). The main clinical manifestations in the dead chicks were kidney swelling, urate deposition on the kidneys, and heavy exudates of flaxen mucus on the bursa of Fabricius (**Figure 3B**). The mental status and appetite in the JS-GD group showed no obvious changes. The organs also exhibited no apparent pathological changes after challenge in the JS-GD group during the observation period.

#### Virus Detection in Organs after Challenge

Reverse transcription–polymerase chain reaction was used for viral RNA detection in sampled tissues after challenge with IBV GD strain. For the control-GD group, virus distribution in different proportions were detected in all organs at 5 and 7 dpc, in addition to the spleen at 7 dpc. For the immunized JS-GD group, the virus was positive only in the bursa of Fabricius of the chicks at 7 dpc. The proportion of positive samples in the JS-GD group was significantly lower than that of the control-GD group. Furthermore, no virus was detected in any organs of the control group (**Table 2**).

**Table 2:** Viral RNA detection for organs and tissues from 9-week-old SPF chickens challenged with IBV strain GD by RT-PCR.

Groups	dpc^a^	Organs and tissues					
		Trachea	Lung	Kidney	Spleen	Glandular stomach	Bursa of Fabricius
Control	5	0/3^b^	0/3	0/3	0/3	0/3	0/3
	7	0/3	0/3	0/3	0/3	0/3	0/3
Control-GD	5	3/3	2/3	1/3	1/3	1/3	3/3
	7	3/3	2/3	3/3	0/3	2/3	2/3
JS-GD	5	0/3	0/3	0/3	0/3	0/3	0/3
	7	0/3	0/3	0/3	0/3	0/3	2/3

*^a^Day post-challenge.*

*^b^Number of positive birds/total number of birds sampled at each time point.*

#### Virus Detection from Laryngeal Swabs after Challenge

As shown in **Table 3**, for all the swabs collected from the larynxes of the unvaccinated chickens at 5 and 7 dpc in the control-GD group, the virus-positive rate was 100%. In contrast, the positive rate for all the JS-GD group samples was 0, while in the control group no viruses were detected in the laryngeal swabs.

**Table 3:** Viral RNA detection for larynxes from 9-week-old SPF chickens challenged with IBV strain GD by RT-PCR.

Group	dpc^a^	
	5	7
Control	0/10^b^	0/10
Control-GD	10/10	10/10
JS-GD	0/10	0/10

*^a^Day post-challenge.*

*^b^Number of positive birds/total number of birds sampled at each time point.*

#### Tracheal Ciliary Activity Grading

**FIGURE 4:**
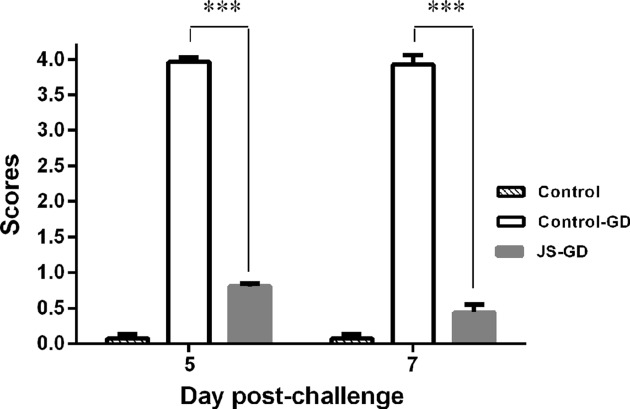
**Trachea ciliostasis scores for 9-week-old SPF chickens experimentally challenged with IBV GD strain.** The degree of ciliary activity inhibition was determined at 5 and 7 dpc in the trachea. The error bar indicates standard deviation. Comparison between two groups was performed using *t*-test. Statistical significance was considered as follows: very highly significant at ^∗∗∗^*P* ≤ 0.001.

We determined the degree of ciliary activity inhibition at 5 and 7 dpc in the trachea. The control-GD group showed a maximum average ciliostasis score of 4, while the average ciliostasis scores for the control and JS-GD groups were below 1 (**Figure 4**). The difference in the level of ciliostasis was extremely significant between the group vaccinated with the JS strain and the unvaccinated group (*P* < 0.001).

#### ELISA Antibody Determination

**FIGURE 5:**
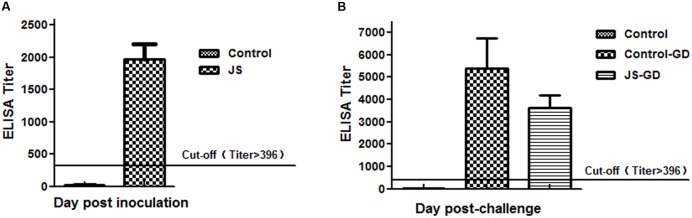
**Seroconversion in chickens experimentally infected with IBV strains. (A)** Mean antibody titers at 21 days post-inoculation (dpi) with the IBV JS strain. **(B)** Mean antibody titers at 14 dpc with the IBV GD strain. Cutoff titer = 396. The error bar indicates standard deviation.

Serum samples were collected for ELISA antibody tests from 21 dpi and 14 dpc. All chicks inoculated with the JS strain showed positive serum antibody responses at 21 dpi and the average antibody titer was 1966.93. And the average titers of the control-GD and JS-GD groups were 5402.60 and 3632.59 at 14 dpc, respectively. Antibodies against IBV were never detected in the control group (**Figure 5**).

## Discussion

Despite the various live-attenuated and inactivated IBV vaccines derived from large numbers of genetically distinct strains in widespread use across the world, IB outbreaks still occur sporadically because there is little or no cross protection between the different IBV serotypes and new variants constantly appear (Cook et al., 2001; Liu et al., 2009; Zhao et al., 2015). Once a new epidemic serotype appears, the protection provided by the existing vaccine strains will greatly decline. The emergence of TW-like strains has been increasing and spreading widely in China during recent years and these TW-like isolates are distantly related to the predominant H120 vaccine and other prevalent strains (Xu et al., 2016). In this study, we determined the pathogenicity of the TW-like IBV strain GD and evaluated the protective efficacy induced by experimental infections with the QX-like JS strain.

The TW-like IBV GD strain was highly pathogenic in SPF chickens, inflicting 100% morbidity and 30–40% mortality. IBV GD caused pale and swollen kidneys, and the renal tubules and ureters were distended with urates. The gross lesions and histopathological changes caused by the GD strain were similar to those described in a report on nephropathogenic IBV strains (Boroomand et al., 2012), thus characterizing GD as a nephropathogenic IBV strain. Additionally, a proportion of the dead chickens in the challenge infection group showed heavy mucus exudates or atrophy in the bursa of Fabricius, a finding similar to the results obtained for other field strains in several previously studies (Toro et al., 2006; Feng et al., 2012). More works need to be performed to clarify this and explore its influence on chickens.

The ciliostasis test is often used to determine the degree of damage to the trachea following growth of IBV in this tissue (Cook et al., 1976; Tarpey et al., 2006). We scored the degree of integrity and the preservation of ciliary movement in the tracheal epithelial cells at 3–10 dpc in the present study after challenge with IBV GD strain. The results revealed that IBV GD induces a severe respiratory disease and lesions in the experimental chickens, and the associated ciliostasis is extremely severe. Consequently, a secondary infection with other pathogens may be acquired more easily when the protective capability of the tracheal cilia is weakened or lost.

The IBV QX-like strain JS has naturally low virulence and shows good protective efficacy against QX-like virulent field strains. Our findings in this study have further revealed that the QX-like IBV JS strain also provided effective protection against TW-like viruses, with no obvious clinical signs or gross lesions, a clear decrease in ciliostasis, and reduced tissue replication rates and viral shedding in the infected chickens.

In summary, our study has revealed that the TW-like IBV GD strain is highly pathogenic in chickens and that the QX-like JS strain provided effective protection against TW-like IBV viruses, indicating that the JS strain may be useful in vaccination programs under field conditions to reduce the economic losses caused by TW-like IBV infections.

## Author Contributions

Conceived and designed the experiments: S-hY, JZ, and G-zZ. Performed the experiments: S-hY, YC, and GX. Analyzed the data: JZ, YZ, and G-zZ. Contributed reagents/materials/analysis tools: G-zZ. Wrote the paper: S-hY, YC, and G-zZ.

## Conflict of Interest Statement

The authors declare that the research was conducted in the absence of any commercial or financial relationships that could be construed as a potential conflict of interest.
